# A Cross-Sectional Audit of Sorghum in Selected Cereal Food Products in Australian Supermarkets

**DOI:** 10.3390/nu14091821

**Published:** 2022-04-27

**Authors:** Cecily Ducksbury, Anita Stefoska-Needham

**Affiliations:** 1 School of Medical, Indigenous and Health Sciences, Faculty of Science, Medicine and Health, University of Wollongong, Wollongong, NSW 2522, Australia; anitasn@uow.edu.au; 2 Illawarra Health and Medical Research Institute, University of Wollongong, Wollongong, NSW 2522, Australia

**Keywords:** sorghum, supermarket audit, snack bar, breakfast cereal, product innovation

## Abstract

Sorghum (*Sorghum bicolor* (L.) Moench) may play a role in mechanisms that elicit favourable health effects. In Australia, sorghum is successfully grown, but it is not widely consumed, and its presence in common food products is unknown. This study examined the utilisation of sorghum in common food products, specifically breakfast cereals and snack bars, in a cross-sectional study of five supermarkets in New South Wales, over a 7-day period in February 2020. Details relating to ingredients, food format, brand, and product name were recorded. Sorghum was present in 6.1% (23/379) of breakfast cereals in a variety of formats, such as extruded shapes, flour, and puffed grain. In 8.7% of these, sorghum was listed as the first ingredient (greatest contribution by weight). Sorghum was utilised in 2% (6/298) of snack bars mainly as puffed sorghum and was listed in the fourth or subsequent position in the ingredient lists for all. ‘Sorghum’ did not appear in the name of any products. In conclusion, this baseline study indicates that sorghum is present in a small proportion of breakfast cereals and snack bars, highlighting the opportunity for greater investment in sorghum food innovation and marketing that would encourage consumer recognition and expand the product range.

## 1. Introduction

Sorghum (*Sorghum bicolor* (L.) Moench) is the fifth most cultivated cereal grain worldwide [1] and is a traditional and often staple food in some arid and semi-arid regions of Africa, Central America, and Asia [2]. In these regions, sorghum is commonly consumed as a key ingredient in flatbreads, porridges, and beer [2], whereas in diets in which sorghum is not traditionally consumed, it is being utilised in a variety of food formats, including alcohols [3], breakfast cereals [4], cake mixes [5], instant meals [6], pretzels [7], popcorn [8], and vegetarian burger patties [9], all with favourable consumer reviews. Depending on factors such as processing, cooking, cooling, food storage, gelatinisation, and cultivar, sorghum may be a rich source of protein, dietary fibre, starch (including slowly digestible starches), non-starch polysaccharides (such as β-glucans), vitamins (notably B-group vitamins), and minerals (including iron and zinc) [10]. Compared with more commonly consumed grains such as wheat, rice, and maize, some cultivars of sorghum have also been shown to have higher levels of polyphenolic compounds such as phenolic acids and flavonoids, which exhibit strong antioxidant, pro-health properties [10,11].

Recent critical appraisals of the evidence base confirm that sorghum may elicit a number of health-promoting effects, including beneficial changes to blood glucose levels, blood lipid levels, and markers of oxidative stress [12,13]. A small number of high-quality studies also suggest that sorghum components are involved in satiety-enhancing mechanisms that may assist with weight management over time and with chronic intake [14,15]. Collectively, these measures represent key risk factors for chronic disease development; hence, there may be potential for regular sorghum consumption, as part of a healthy diet, to assist in disease prevention. This is of paramount importance in Western societies, including those in Europe, the United States, and Australia, where obesity-related chronic diseases are a major public health burden [16]. In addition to its potential beneficial health effects, there is an opportunity for sorghum to serve as a sustainable source of human nutrition, which is of global significance in the face of environmental concerns related to climate variability [17]. Dubbed a ‘life-saver cereal’ in drought-prone regions of the world where food is in short supply, sorghum is widely grown in soils in which production of other cereals, such as wheat, may fail [18,19]. This superior adaptability is in part due to a more extensive and deeper penetrating root system, as well as leaf rolling for moisture conservation [20]. In Australia, despite high local grain production levels [21], sorghum is not part of the traditional Australian diet and is used predominantly for livestock feed and export markets. Hence, the commercialisation of sorghum food products has been relatively slow [17,20]. According to Stefoska-Needham and Tapsell [17], an interplay of factors may have contributed to this aspect, including poor recognition of the grain as human food, both by producers and consumers; production capacity oriented towards the animal feed market limiting the availability of human-grade sorghum in the food supply; and lack of high-level evidence supporting the use of the grain as a health food. A positive shift in uptake of sorghum as an alternative grain source will require a change in attitude towards the grain by multiple stakeholders in the value chain, including food manufacturers, grain growers, grain advocacy groups, dietitians and nutritionists, researchers, and—most importantly—consumers. Furthermore, recent world events, including war and its associated political sanctions, and the COVID-19 pandemic, have produced notable disruptions to global supply chains in a wide range of goods, including food [22]. These events are causing governments, industries, and commercial companies to rethink where they conduct business and what their target may be. This effect may also present a driving force for locally grown sorghum to be used in human food applications.

Currently, the prevalence of sorghum in the Australian human food supply is unknown, though it is estimated to be less than wheat, which is the most commonly consumed grain [23]. Awareness of the range of available products and their market position is, therefore, an important step in understanding the commercial importance of sorghum in the Australian human food supply and its potential adoption pathway. Hence, the aim of the present study is to benchmark, for future monitoring, the presence of sorghum in commonly consumed food products, notably breakfast cereals and snack bars, in a cross-sectional study of major Australian supermarkets.

## 2. Materials and Methods

A cross-sectional supermarket audit was conducted in the Illawarra region of New South Wales to evaluate the use of sorghum as an ingredient in breakfast cereal and snack bar products available to consumers in the region. This audit is the first of a series of intended audits planned for the future.

### 2.1. Product Selection

In Australia, the breakfast cereal and snack bar markets are extensive [24,25], comprising a broad range of products, both in terms of types and numbers. For this reason, the breakfast cereal and snack bar categories were chosen for inclusion in this first-time Australian audit of sorghum in food products. Breakfast cereals are consumed by over a third of all Australians (36.1%), and snack bars are consumed by 16% of children and 7.5% of adults [23]. Sorghum presents rheological challenges for bread making, and it was previously anticipated that it would not be used extensively; hence, bread was not included in the audit [26]. Based on previous Australian audits [27,28,29], it was estimated that the breakfast cereal product range would consist of approximately 200–330 products and approximately 165 cereal bars (excluding nut/seed bars) [27,30]. Hence, to fit within the scope of this audit, only breakfast cereals in the breakfast cereal and health food sections of the supermarkets were included, as well as all snack bars in the snack bar and health food sections. Snack bars located in the specialty nutritional supplement section of supermarkets (such as specialised protein bars) were excluded.

### 2.2. Supermarket Selection

A food product audit was conducted on three major Australian supermarket chains across the Illawarra region of NSW: Woolworths (37.6% market share), Coles (29.1% market share), and Aldi (9.9% market share) [31]. Supermarket selection was determined by market share, sociodemographic status (as per Socio-Economic Indexes for Areas (SEIFA) [32]), and other supermarket audits conducted in the Illawarra [29,33]. Three Woolworths stores (located in Bulli, Shellharbour, and Unanderra), one ALDI supermarket, and one Coles supermarket (both in Wollongong) were included, maximising exposure to a varied product range [31]. To fit within the scope of this study, smaller supermarkets and specialty stores, such as health food stores, were not included in the audit. Prior to the audits being conducted in-store, permission to collect data was received from the management of all supermarkets involved.

### 2.3. Data Collection

Data collection was carried out over a 7-day period in February 2020. Products included in the study were all breakfast cereals available in the breakfast cereal and health food section of the supermarkets and all snack bars available in the snack bar and health food sections, on the day of data collection. Data collection involved photographing the front of the box, nutrition panel, and ingredient list of all products meeting the above criteria. If the same product came in multiple packaging sizes, only one size (the largest) was recorded to avoid duplication of the same formulation. Similarly, if the same product was available at multiple store locations, the product was only included once for analysis.

From the photographs, the following data were extracted: brand, product name, and supermarket source. The ingredients panel was then evaluated to identify if sorghum was an ingredient. If sorghum was present in the formulation, the ingredients list was recorded, as were the sorghum format (e.g., flour) and the position of sorghum in the ingredients list. Ingredients in Australian products are listed in descending order of weight proportion in a formulation; hence, the position of sorghum in the ingredients list is reflective of the quantity of sorghum in the formulation (Food Standards Australia New Zealand (FSANZ) Standard 1.2.4) [34].

### 2.4. Data Analysis

Data were descriptively analysed using Microsoft Excel 365 (v 16.0, Washington, DC, USA) and was presented as counts and percentages to evaluate the presence of sorghum across categories, the proportion of sorghum across categories, the format of sorghum included in products (e.g., sorghum flour), the level of processing (refined or whole-grain sorghum), and finally, to evaluate what proportion of products containing sorghum were gluten-free, marketed for children (as determined by the presence of the term ‘children’ or similar, or cartoon images on the packaging inferring the target is children), or marketed in the health food sections of stores. Product brands and names are not identified in this report.

Data analysis was completed separately for breakfast cereals and snack bars. Breakfast cereals were categorised into eight groups based on formulation characteristics of the cereals: bran sticks, bubbles, puffs and flakes (e.g., corn flakes, rice puffs), clusters (e.g., clusters of oats nuts, and fruits), extruded shapes (e.g., hoop shape cereal), flaked biscuit (e.g., whole wheat biscuit), granola (e.g., toasted oats with fruits and seeds), muesli (e.g., untoasted oats with fruits or seeds), and porridge (e.g., rolled oats). Snack bars were categorised into nine groups based on formulation characteristics of the bars: biscuit-style bars(e.g., hard, flat bars with chocolate filling), cake-style bars (e.g., soft, chocolate-fudge-flavoured bars), filled bars (e.g., soft bars with fruit-based filling), muesli/cereal bars (e.g., bars containing oats, rice puffs, wheat, and fruits), nut/seed bars (e.g., peanut and almond bars), oat-bake-style bar (e.g., soft-baked bars with oats and fruit), pressed bars (e.g., apricot puree based bars), puff/bubble bar (e.g., chocolate-flavoured rice puff bars) and rolled-oat-style bars (e.g., rolled oat bars with chocolate chips).

### 2.5. Data Quality

Data quality was independently checked by a second researcher (ASN). A randomised selection of 10% of products from each database was reviewed for accuracy of data input, classification, and sorghum contents.

## 3. Results

A total of 677 products were audited and analysed in this study, of which 379 were breakfast cereals, and 298 were snack bars.

### 3.1. Breakfast Cereal Audit

Records of 379 breakfast cereal products were identified for analysis (Table 1). Of the eight subcategories of breakfast cereals identified, porridge-style cereals were the most common (21.9%), followed by bubbles, puffs, and flakes (21.1%), and muesli (19.5%). In total, 23/379 (6.1%) of analysed breakfast cereals included sorghum as an ingredient in their formulations. These sorghum-containing cereals varied according to the following subcategories: extruded shapes (*n* = 7); flaked biscuits (*n* = 5); bubbles, puffs, and flakes (*n* = 4); muesli (*n* = 3); oats/porridge (*n* = 3); and clusters (*n* = 1) (Table 1).

Sorghum was stated first in the ingredient list in 8.7% of surveyed sorghum containing breakfast cereal products, second in 39.1% of products, and third or beyond third (≥3) in 26.1% of products, respectively. The format of the sorghum used in the products was most listed as ‘whole-grain sorghum flour’ (*n* = 10), ‘whole-grain sorghum’ (*n* = 7), ‘sorghum crisps’ (*n* = 3), ‘sorghum flour’ (*n* = 2) and ‘puffed sorghum’ (*n* = 1). Whole-grain sorghum was listed in 17/23 products (73.9%). In the remaining products, it was not specified if the grain was refined or whole grain. Sorghum was more frequently included as an ingredient within extruded shaped cereals and flaked breakfast biscuits formulations (22.7% and 20.6%, respectively), compared with the other breakfast cereal subcategories for which sorghum was identified.

**Figure 1:**
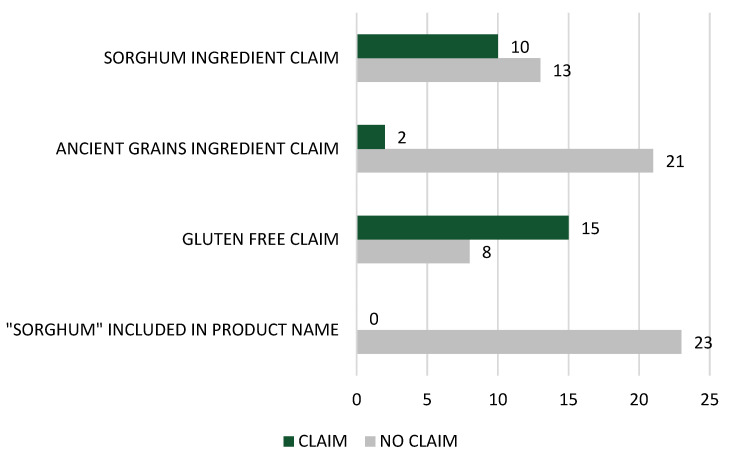
Key front-of-pack claims from sorghum-containing breakfast cereals.

A total of 15/23 (65.2%) sorghum-containing cereals were classified as gluten-free, and 6/23 (26.1%) were marketed to children. Of the breakfast cereal products targeting children, all were gluten-free and used whole-grain sorghum. Of the 23 sorghum containing breakfast cereals identified in this research 12/23 (52.2%) were found in the specialty health food section of the supermarket. Sorghum was not mentioned in the title of any breakfast cereal products; however, sorghum was included as a product descriptor in 10/23 breakfast cereal products (for example, ‘with puffed sorghum’) (Figure 1).

### 3.2. Snack Bar Audit

Records from 298 snack bar products were identified for analysis (Table 2). Of the nine categories of snack bars surveyed, nut/seed-based bars were the most common (36.2%), followed by rolled-oat-style bars (18.5%) and pressed bars (16.1%). In total 6/298 (2.0%) of bars contained sorghum. Of these, three were categorised as muesli/cereal bars and the remainder as puff/bubble-style bars. The format and position of sorghum within these bars did not vary. All six bars contained ‘puffed sorghum’ which was in the *≥3* position in the ingredients list. No bar specified if the puffed sorghum was whole grain. All snack bars containing sorghum were labelled as gluten-free, and 50% of the bars were marketed towards children. All snack bars containing sorghum were located in the health food section of the store. Sorghum was not mentioned in the title of any snack bar products, nor was sorghum mentioned as a descriptor on front-of-pack labels. Furthermore, no sorghum-based snack bar products used the ‘ancient grain’ descriptor on their front-of-pack labels.

## 4. Discussion

In this Australian-first study, the prevalence of sorghum as an ingredient in selected grain-based food products was evaluated. In a cross-sectional, targeted audit of commercial products available in supermarkets, sorghum was identified as an ingredient in 6.1% of breakfast cereals and 2.0% of snack bars. Although the extent to which sorghum is present in other food product categories is unknown at this point in time, these initial findings are consistent with worldwide consumption data that indicates sorghum food product innovations are developing and diversifying across human food supply chains in countries where sorghum has not been traditionally consumed, such as Australia and the United States [35]. Mapping the utilisation of sorghum in products in the manner described in this study is important, as it may serve as a benchmark to monitor the future trends in sorghum utilisation for human food production and thereby provide useful insights to guide future sorghum food product innovations and investment.

The breakfast cereal category represents an important commercial opportunity for sorghum innovations, especially in Australia, where the breakfast cereal market is valued at over AUD 1.9 billion annually [24], accounting for 44.1% of whole-grain intake in children (aged 2–18 years) and 46.2% in adults [36]. In the present study, sorghum-containing products were identified across most breakfast cereal categories. This finding reflects broader consumer acceptability and product innovation research, which indicates that sorghum can be successfully integrated into various breakfast cereal formulations, including granolas, flakes, extruded puffs, and flaked biscuits [14,37,38,39]. In the sorghum-containing breakfast cereals identified, extruded-shaped cereals were the most common, followed by flaked biscuits. Extrusion technology has been frequently utilised for the development of sorghum breakfast cereals and snacks due to the desirable textures, shapes, and crunch that may be achieved by the process [40,41]. In products utilising pigmented varieties of sorghum, in particular, high levels of polyphenolic components with high antioxidant activities have been reported, following extrusion processing [37,38]. Similarly, following the flaking process, during which whole-grain red sorghum is soaked, air-dried, rolled, and toasted, flaked breakfast biscuits have been shown to retain high levels of antioxidant compounds, dietary fibre, and slowly digestible starch [14].

In the present study, whole-grain sorghum was identified in the top three ingredients of almost three-quarters of sorghum-containing breakfast cereals reviewed in this study; however, the grain was more commonly incorporated as a non-characterising ingredient among a combination of other cereals such as barley, oats, rice, and wheat. Only one breakfast cereal product contained sorghum as the primary ingredient—a flaked biscuit made of 96% whole-grain sorghum. Research demonstrates sorghum may be successfully integrated into various breakfast cereal formulations as the primary ingredient, and in most cases, these formulations may include more than 90% whole-grain sorghum and maintain positive consumer acceptance [14,37,38,39].

Compared with breakfast cereals, the use of sorghum in snack bar samples reviewed in this study was less frequent and featured in smaller quantities, which is likely to be influenced by a number of factors. Firstly, the Australian breakfast cereal market is significantly larger (AUD 1.9 billion) than that of the grain-based snack industry in which muesli and cereal snack bars are included (AUD 58 million) [24,25]. Further, the current snack bar product range appears to be dominated by nut/seed-style bars (36.2%) and oat-based muesli bars (18.5%), with only a fraction of bars containing alternative whole grains such as sorghum. This may present reduced incentives for grain-based snack manufacturers to explore innovations including sorghum. In addition, compared with sorghum-based breakfast cereals [14,37,38], experimental research exploring the development and consumer acceptability of snack bars containing sorghum is limited, with a recent exception, where a study demonstrated favourable consumer acceptability and reasonable shelf life of a gluten-free bar containing a combination of popped and extruded sorghum [42]. Therefore, the opportunity to add sorghum to the formulation of nut/seed-style and oat-based bars does not appear to be of interest to food manufacturers currently, possibly because nut-/seed-/oat-free bars are less popular with consumers [43]. Understanding consumers’ desires and preferences for new grains such as sorghum in snack bar formulations offers further insight to help direct the future direction of product innovation.

Overall, the marketing of sorghum in breakfast cereals and snack bars appears to be limited, compared with other grains such as wheat and oats. Marketing is a key strategy that can be used to educate consumers about sorghum’s competitive advantage, beyond just its gluten-free attribute which currently dominates the labels of sorghum-containing products (all snack bars and almost two-thirds of all breakfast cereals were labelled ‘gluten-free’). Explicit use of the word ‘sorghum’ on front-of-pack labelling and within product names is a key step change, especially given that this study did not identify any products that contained the word ‘sorghum’ in their product name. Stating ‘sorghum’ alongside other trending descriptors, such as ‘ancient grains’ or ‘gluten-free’, may also be strategic to increase recognition of the grain [4,24,44]. Sorghum’s ‘whole-grain’ attribute should also be a key leverage point for marketing, especially because consumers perceive whole grains to be important for their health, and they associate whole-grain usage with high fibre content and less processing [45]. Furthermore, the evidence base indicates that whole-grain intake is associated with a reduction in the risk of common chronic diseases, including cardiovascular disease, type 2 diabetes, and some cancers, as well as a reduction in the risk of overweight and obesity [46,47,48], and this is also the case for regular whole-grain sorghum consumption [12,13,14]. Increasingly, food manufacturers acknowledge these consumer beliefs by routinely promoting ‘whole grains’ on front-of-pack labelling. In fact, whole-grain content claims on breakfast cereals have doubled since 2013, alongside a modest increase in products containing at least 8 g of whole grains per serve [49]. In the case of new sorghum product innovations, marketing whole-grain content will be an important strategy to influence purchasing decisions and increase sorghum per capita consumption.

We encourage marketers and other stakeholders to promote sorghum as an appealing grain alternative that offers favourable health benefits but is also more sustainably produced and has claims to provenance, especially in Australia and the United States, where production rates are high [1]. Both environmental sustainability and provenance have been shown to be powerful consumer drivers in recent years [50,51] and should be leveraged to influence purchasing decisions towards sorghum-based products. Additionally, from a marketing perspective, a key observation in this study was that a quarter of the sorghum-containing breakfast cereals were targeted towards children. These children’s products used extruded shaped formulations made from whole-grain sorghum, exploiting the ability of the grain to be shaped into crunchy shapes appealing to children, such as rings and spheres. This food format is commonly used in many popular Australian children’s cereals and cereal bars [52], with cereal bars marketed to children representing a promising opportunity for future sorghum innovation in particular.

More broadly, the results of this study may serve as a useful guide for innovators and researchers interested in designing new sorghum foods. According to Rodger’s diffusion of innovation (DOI) theory [53], the uptake of ideas is a gradual process, and there are five factors that affect the adoption of new innovations: relative advantage (to previous innovations), compatibility, complexity (ease of use), trialability, and observability [53,54]. For the successful integration of sorghum foods into a rapidly growing Australian health and functional food market [25,26], high-quality research encompassing both the environmental and health effects of sorghum consumption is needed to raise awareness of the grain’s potential advantage and value in the human food supply. In this regard, investment in food science research and clinical trials through partnerships between academia and industry will be paramount [17,55]. More product and consumer research studies are needed to enhance the quality of sorghum products to ensure they are equal to or even superior to competitor products [17,26]. Initiatives and research that involve sorghum growers to encourage diversification of sorghum production into industries other than feed for livestock are also important for the future, and these may encompass economic, environmental, and agronomic aspects of innovation [56]. Manufacturer action to label sorghum usage on front-of-pack labels is also an important step to increasing consumer recognition of the grain. Furthermore, specialised research into specific markets would be beneficial to increase manufacturer awareness of potential opportunities for sorghum usage. For instance, conducting research that compares the nutritional and functional value of *sorghum-containing* versus *non-sorghum-containing* cereal products appealing to children would help to identify points of difference that could be leveraged to market sorghum to this consumer group, including their parents who have the purchasing power. Finally, increased advocacy for the grain by nutrition groups such as the Grains and Legumes Nutrition Council [57] would assist to improve consumer and healthcare provider recognition of the grain and thereby encourage greater sorghum consumption. Overall, a commitment to product innovation involving a range of stakeholders along the value chain, such as researchers, producers, marketers, government, dietitians, and consumers, is required to encourage greater utilisation of sorghum human food production and to expand the range of sorghum-containing products available to consumers.

### Study Strengths and Limitations

The seminal nature of this research is a key strength of this study which aims to evaluate the presence of sorghum as an ingredient in commonly consumed Australian food products. As part of a larger project, only breakfast cereals and snack bars were audited in this study, representing phase one. In future phases, the product range should be expanded to other categories, enabling a more accurate measure of sorghum’s prevalence in a wider range of food products. This study was strengthened by the large sample size of products included in the analysis stemming from the large representation of breakfast cereals and snack bar products available in Australian supermarkets. Attention to processes that ensure the study methodology is repeatable is also a strength of the study, particularly to enable the intended ongoing monitoring of sorghum in products in future research. For ethical reasons and in agreement with store management, no comparisons between stores or brands could be made within this project; hence, this may be perceived as a limitation. This research was limited to major Australian supermarkets as the intent of this research was to create a benchmark of the prevalence of sorghum in mainstream food products, and to this end, smaller health food stores were not included. This could be considered a limitation, as these small to medium enterprises are more likely to be early adopters of products that have been innovated with ‘novel’ health foods ingredients, compared with larger companies [24,44]. Hence, the next phases should include audits of smaller suppliers and specialty health food stores. Finally, the percentage quantity of sorghum used within the product formulations was not disclosed as the Australian New Zealand Food Standards Code only requires the labelling of the ingredients making up a food product and not the quantity used in the formulation, unless the ingredient is stated on the packaging (FSANZ Standard 1.2.10) [58], which may be perceived as a limitation.

## 5. Conclusions

Sorghum is utilised as an ingredient in a small number of breakfast cereal and snack bar products available to consumers in major Australian supermarkets. This major finding, together with results of future planned audits across more food categories, will offer a more accurate representation of the prevalence of sorghum in the Australian human food supply over time. This has significance for food manufacturers seeking to develop and market new sorghum products that exploit the health-promoting attributes of grain constituents and the sustainable production of the crop, as well as to grain advocates (such as nutritionists), whose awareness of what products are available to consumers on supermarket shelves, including their composition, is important. Last but not least, ongoing monitoring of sorghum in foods may help to inform sorghum growers of existing value-adding product opportunities that may unlock potential revenue streams and raise profit margins, in conjunction with increasing per capita consumption, representing an important step for the industry broadly.

## Figures and Tables

**Table 1:** Prevalence of sorghum ingredients in breakfast cereals surveyed in the supermarket audit.

Breakfast Cereal Category	Products Examined		Products Containing Sorghum		
	*n*	Percentage of Total Products Surveyed (%)	*n*	Percentage of Products Containing Sorghum within Subcategory (%)	Proportion of Total Products Surveyed Containing Sorghum (%)
Bran Sticks	5	1.3	0	0.0	0.0
Bubbles, Puffs, and Flakes	80	21.1	4	5.0	1.1
Cluster	41	10.8	1	2.4	0.3
Extruded Shapes	34	9.0	7	20.6	1.8
Flaked Biscuit	22	5.8	5	22.7	1.3
Granola	40	10.6	0	0.0	0.0
Muesli	74	19.5	3	4.1	0.8
Porridge	83	21.9	3	3.6	0.8
Total	379	100	23		6.1

**Table 2:** Prevalence of sorghum ingredients in snack bars surveyed in the supermarket audit.

Snack Bar Category	Products Examined		Products Containing Sorghum		
	*n*	Percentage of Total Products Surveyed (%)	*n*	Percentage of Products Containing Sorghum within Subcategory (%)	Proportion of Total Products Surveyed Containing Sorghum (%)
Biscuit-Style Bars	2	0.7	0	0	0
Cake-Style Bars	8	2.7	0	0	0
Filled-Style Bars	13	4.4	0	0	0
Muesli/Cereal Bars	18	6.0	3	16.7	1.0
Nut/Seed-Style Bars	108	36.2	0	0	0
Oat Bake-Style Bars	32	10.7	0	0	0
Pressed Bars	48	16.1	0	0	0
Puff/Bubble-Style Bars	14	4.7	3	21.4	1.0
Rolled-Oat-Style Bars	55	18.5	0	0	0
Total	298	100.0	6		2.0

## Data Availability

The datasets generated during the current study are not publicly available, as they contain commercially sensitive information.
